# Fearing Forgetting: Dementia-Related Anxiety and Its Effects on Caregivers, Mental Health, and Support Strategies

**DOI:** 10.1093/geroni/igaf122.455

**Published:** 2025-12-31

**Authors:** Sabine Lohmar, Allie Peckham

## Abstract

Alzheimer’s disease and related dementias (hereafter “dementia”) are among the most feared diseases (Awang et al., 2018). With increased exposure to dementia (e.g., caregiving; media portrayals), individuals can develop dementia worry (DW) or dementia-related anxiety (DRA), which can range in strength from mild worry to severe anxiety or phobia (Kessler et al., 2012). This symposium will explore various aspects of DW/DRA, focusing on how it impacts caregivers and those at risk for dementia and its impact on psychosocial outcomes. Sabine Lohmar, M.S., will introduce the topic with results from a systematic review on the relationship between DW and depression, highlighting at-risk groups and implications for dementia treatment. Dr. Maxfield will discuss the interpersonal consequences of DRA and association with anticipated suicidal and death ideation. Dr. Lundquist will present on her work utilizing a quantitative measure of anxiety about Alzheimer’s disease with a focus on implications for improving support of individuals with DRA. Dr. Baker will share findings from a series of Community Advisory Board meetings, exploring how DRA might help bereaved dementia caregivers adopt health behaviors that could reduce their own dementia risk, inspired by caregiver fears of developing dementia. This symposium explores DW and DRA through diverse methodological approaches, including systematic review, qualitative and quantitative research, community engaged inquiry, and clinical assessment to explore the impact of DRA/DW in caregivers and at-risk individuals.

